# Self-reported Gastrointestinal Side Effects of Antifibrotic Drugs in Dutch Idiopathic Pulmonary Fibrosis patients

**DOI:** 10.1007/s00408-019-00260-1

**Published:** 2019-08-22

**Authors:** V. L. J. Proesmans, M. Drent, M. D. P. Elfferich, P. A. H. M. Wijnen, N. T. Jessurun, A. Bast

**Affiliations:** 1 Venlo Campus, Maastricht University, Venlo, The Netherlands; 2 ILD Center of Excellence, Department of Pulmonology, St. Antonius Hospital, Koekoekslaan 1, 3435 CM Nieuwegein, The Netherlands; 3 Department of Pharmacology and Toxicology, FHML, Maastricht University, Maastricht, The Netherlands; 4 ILD Care Foundation Research Team, Ede, The Netherlands; 5 Central Diagnostic Laboratory, Maastricht University Medical Centre, Maastricht, The Netherlands; 6 Netherlands’ Pharmacovigilance Centre Lareb, ’s Hertogenbosch, The Netherlands

**Keywords:** Antifibrotic drugs, Idiopathic pulmonary fibrosis, IPF, Nintedanib, Pirfenidone, Side effects, Treatment

## Abstract

**Purpose:**

Idiopathic pulmonary fibrosis (IPF) is an inexorably progressive disease, which has a great impact on patients’ lives. Pirfenidone and nintedanib are approved and recommended antifibrotic drugs for patients with IPF. The aim of this study was to evaluate self-reported gastrointestinal side effects of antifibrotic drugs in 176 Dutch IPF patients**.**

**Methods:**

A cross-sectional web-based anonymous survey about complaints and side effects was conducted among IPF patients in the Netherlands. Logistic regression was used to quantify whether pirfenidone and nintedanib caused complaints of nausea, vomiting, diarrhoea, appetite loss, weight loss or loss of taste or smell perception.

**Results:**

The questionnaire was completed by 176 IPF patients, 71 of whom used pirfenidone and 85 nintedanib, while 20 patients did not use any antifibrotic drugs. Nintedanib users reported complaints of diarrhoea, vomiting, weight loss and loss of appetite (*p* < 0.01). Nausea was a significant adverse reaction (*p* < 0.05). Pirfenidone caused increased appetite loss (*p* < 0.01) and the risk of weight loss (*p* < 0.05). The increase in loss of appetite and weight loss did not differ significantly between the two drugs.

**Conclusion:**

The current study showed that nintedanib causes a significant increase in diarrhoea, vomiting, weight loss and loss of appetite, while pirfenidone led to loss of appetite. Our results suggest new avenues regarding dietary recommendations for IPF patients.

## Introduction

Idiopathic pulmonary fibrosis (IPF) is a serious, inexorably progressive disease, which usually affects middle-aged and older adults. While IPF is by definition “idiopathic” (i.e. of unknown cause), the list of potential fibrogenic triggers that have been associated with IPF includes smoking, chronic microaspiration of gastric content and chronic infection. IPF varies from person to person. In some cases, fibrosis develops quickly, while in others, the process is much slower and the disease remains stable for years. It carries a 5-year survival rate of approximately 20%, which is worse than that of several types of cancer [1, 2]. Although IPF is the first or second most commonly encountered form of interstitial lung disease (ILD) (range 17–86%), its overall incidence and prevalence are unclear. Published incidence rates have ranged from 0.6 to 17.4 per 100,000 person years. To date, there is no cure for IPF. In addition to other care options endorsed by the ATS guidelines, including pulmonary rehabilitation, long-term oxygen therapy, lung transplantation and antacid therapy, new antifibrotic drugs have recently become available [3]. Pirfenidone and nintedanib, two compounds with antifibrotic properties and pleiotropic mechanisms of action, have consistently proven to be effective in reducing functional decline and disease progression in IPF, and have been approved as standard of care worldwide [3, 4]. Despite substantial differences in the mechanism of action of these two compounds, their treatment effect is strikingly similar, reducing the decline of forced vital capacity (FVC) by approximately 100 mL/year. Individual treatment options should therefore be discussed with each newly diagnosed IPF patient, considering not only the potential benefits but also the side effects, which are not completely identical for these two drugs.

A chronic condition such as IPF may have a substantial impact on patients’ quality of life (QoL) [5], and the same is true for the possible side effects of drugs used to treat this progressive disorder. Common side effects of both drugs include nausea, diarrhoea, weight loss and loss of appetite. Pirfenidone is also known to cause changes in taste and smell perception [6], while similar effects have not been reported for nintedanib. These side effects are regularly reported by patients, but relatively little is known about their real prevalence. We therefore studied the self-reported side effects of antifibrotic drugs in a Dutch sample of IPF patients**.**

## Materials and Methods

### Study Design

In cooperation with the Dutch Pulmonary Fibrosis Patient Society (Longfibrosepatiëntenvereniging Nederland), the ild care foundation has designed a questionnaire about side effects of antifibrotic drugs. This questionnaire includes questions about their disease and any problems these patients may have experienced regarding the use of antifibrotic drugs as well as other medication. In addition, it concerns the burden of disease and the symptoms experienced by patients with IPF. Respondents were asked to complete the questionnaire even if they had never experienced any problems with drug use. The questionnaire was used in a cross-sectional web-based anonymous survey, conducted from June 2018 to October 2018 among a sample of IPF patients in the Netherlands.

This study was performed in accordance with the Declaration of Helsinki and its amendments.

### Study Subjects and Procedure

The overall study sample included IPF patients who were known at the outpatient clinic of the ILD Center of Excellence of the St. Antonius Hospital, Nieuwegein, the Netherlands and/or who were members of the Dutch Pulmonary Fibrosis Patient Society. All subjects had been diagnosed with IPF by a multidisciplinary team according to international guidelines [7]. Patients were recruited without incentives, since the survey was anonymous. All patients had sufficient command of the Dutch language and internet access. The survey was developed using the online questionnaire tool *Surveymonkey* (www.surveymonkey.com). The questions concerned the burden of disease and symptoms experienced by the patients with IPF. Further questions concerned demographics (gender, age, duration of IPF) and the use of medication. Those patients who did not use antifibrotic drugs were considered as controls.

### Statistical Analysis

All statistical analyses were performed using R version 3.5.2, retrieved from the R Foundation for Statistical Computing [8]. To test the adverse effects of pirfenidone and nintedanib, the variables nausea, vomiting, diarrhoea, weight loss, appetite loss and loss of taste or smell perception were evaluated using logistic regression analysis with a logit link. Drug use was included as an explanatory variable, with three factors: pirfenidone (*n* = 71), nintedanib (*n* = 85) and non-drug users (*n* = 20). A correlation matrix (Table 4 in Appendix) was used to select which of the covariates of age, gender, smoking, time since diagnosis, BMI, antacid use, vitamin D, vitamin K and multivitamin supplementation should be included. If a covariate correlated with any of the adverse side effects from a significance level of *p* ≤ 0.05, it was included in the final model.

## Results

Table 1 shows demographic and clinical data from 176 Dutch patients suffering from IPF, 20 of whom did not use any antifibrotic drugs (Group 1). Pirfenidone was used by 71 patients (Group 2) and 85 patients used nintedanib (Group 3). The non-drug users (Group 1) included significantly fewer men (*p* < 0.01) than the drug users (Groups 2 and 3). Other factors did not differ significantly between these groups (Table 1). The antifibrotic drug users were not suffering from any substantial gastrointestinal comorbidities before the start of their antifibrotic treatment. Of those who did not use any antifibrotics, 10% suffered from gastrointestinal comorbidity. In five cases, gastrointestinal side effects could be assumed to be related to concomitant drugs used besides the antifibrotic drugs (3 × metformin and 2 × an antidepressant). However, we did ask the participants explicitly whether their complaints had started after the initiation of the antifibrotic drugs. All but one thought there was a clear relation between starting the antifibrotic drug and the development of the complaints, and that these complaints were not attributable to other drugs they might have used. Though, it should be acknowledged that the effect of concomitant drug use can never be excluded completely.

**Table 1:** Summary of the demographic and clinical data of the three idiopathic pulmonary fibrosis (IPF) patient groups

	Group 1 non-drug users	Group 2 Pirfenidone users	Group 3 Nintedanib users
Number	20	71	85
Age (range, min–max), years	63 (35–79)	70 (43–83)	68 (46–80)
Gender, male %	55*	81.7	78.8
Smoker, yes/no/former %	0/45.0/55.0	1.4/52.1/46.5	3.5/35.3/61.2
Time since diagnosis, years	2.7 ± 0.7	2.7 ± 0.7	2.1 ± 0.7
Having used medication longer than 12 months %	NA	68.1	56.6
Oxygen use, %	31.6	45.5	45.1
BMI (kg/m^2^)	27.2 ± 3.6	26.4 ± 3.6	26.6 ± 3.8
Vitamin D, yes %	50	32.4	37.6
Vitamin K, yes %	25	15.5	18.8
Multivitamin, yes %	15	11.1	11.8
Antacid, yes%	78.9	73.2	71.8

Data are expressed as mean ± SD or percentage if appropriate

*BMI* body mass index, *NA* not applicable

^*^p value < 0.01 group 1 vs 2 + 3

**Fig. 1:**
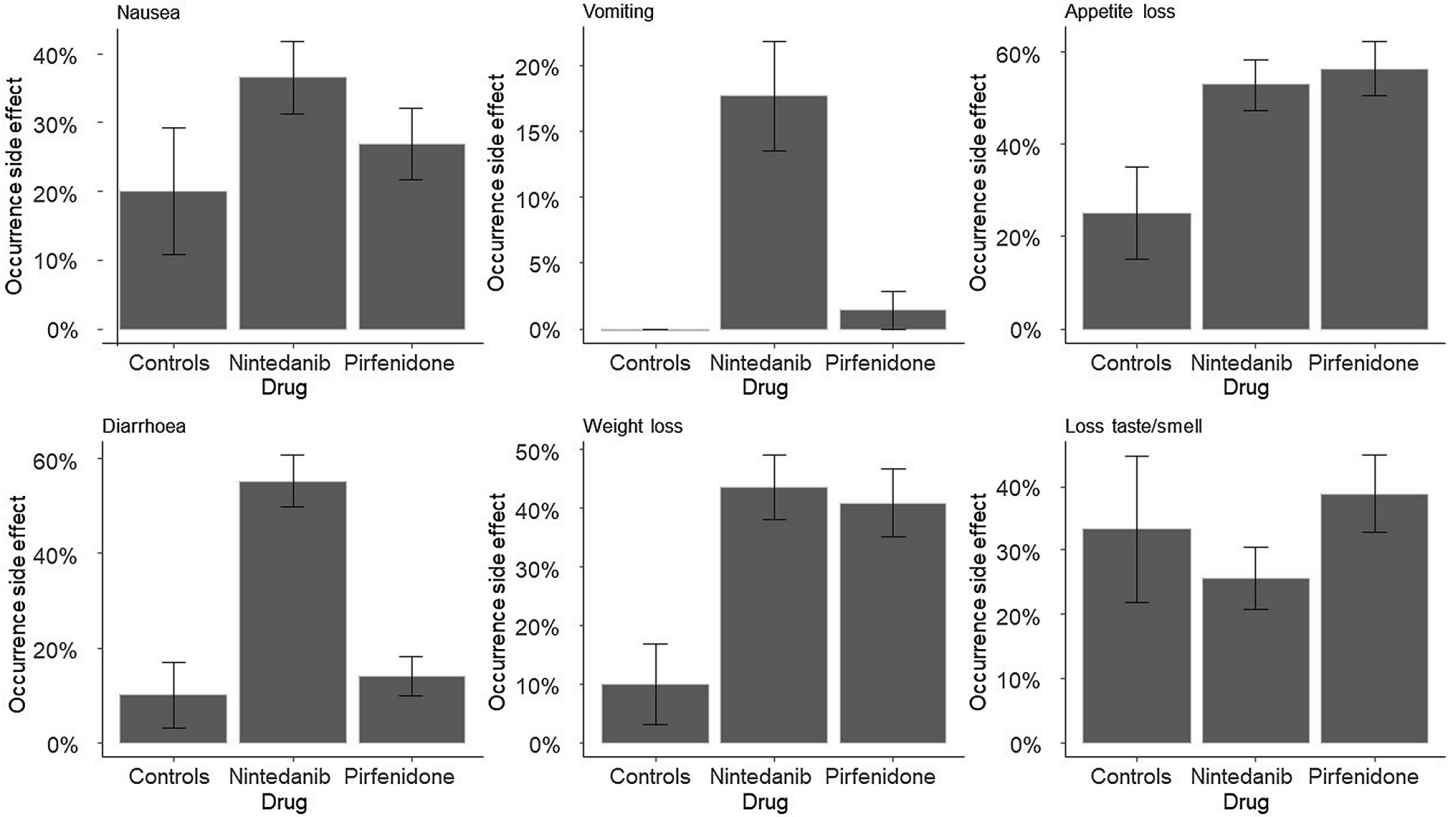
Complaints among IPF patients using nintedanib, pirfenidone or neither (controls). *Value differs significantly from controls (*p* < 0.05). ^#^Value differs significantly from other drug group (*p* < 0.05)

Figure 1 shows the side effects among the three IPF patients groups.

Nintedanib users reported significantly more diarrhoea and weight loss than Group 1 (controls: non-drug users). Because none of the respondents in Group 1 reported vomiting, it was not possible to statistically test for a direct effect. However, there was a significant difference between pirfenidone and nintedanib users in the incidence of vomiting, which was higher among nintedanib users.

Pirfenidone users were more likely to suffer weight loss than subjects in Group 1.

Table 2 shows the effect of both drugs on the occurrence of side effects, taking correlated covariates into account (Table 4 in Appendix).

**Table 2:** Occurrence of side effects among drug users

	Coefficient	Std. Error		*Z* value	*P* value
Nausea					
Intercept	− 0.62	0.61		− 1.03	0.30
Nintedanib	1.51	0.68		2.24	0.02*
Pirfenidone	1.03	0.69		1.50	0.13
Gender	− 1.84	0.42		− 4.39	< 0.01*
Diarrhoea					
Intercept	− 2.20	0.75		− 2.95	< 0.01*
Nintedanib	2.41	0.78		3.10	< 0.01*
Pirfenidone	0.39	0.82		0.48	0.64
Appetite loss^a^					
Intercept	− 1.48	0.71		− 2.10	0.036*
Nintedanib	2.34	0.72		3.24	< 0.01*
Pirfenidone	2.50	0.73		3.42	< 0.01*
Gender	− 1.69	0.49		− 3.46	< 0.01*
Antacid	0.92	0.38		2.42	0.016*
Weight loss^a^					
Intercept	− 0.89	1.62		− 0.55	0.58
Nintedanib	2.08	0.8		2.59	< 0.01*
Pirfenidone	1.98	0.81		2.43	0.015*
BMI	− 0.11	0.05		− 2.09	0.036*
Vit.D	0.8	0.36		2.24	0.025*
Antacid	1.37	0.44		3.08	< 0.01*
Loss of taste or smell	Perception^b^				
Intercept	− 2.25	0.95		− 2.37	0.018*
Nintedanib	0.32	0.66		0.48	0.63
Pirfenidone	1.00	0.67		1.50	0.13
Gender	− 1.26	0.44		− 2.87	< 0.01*
Time since diagnosis	0.46	0.26		1.75	0.080
Antacid	1.06	0.46		2.3	0.021*

^a^1 observation deleted due to missing value

^b^10 observations deleted due to missing values

*Significant influence

Nintedanib (*p* = 0.02) was associated with nausea, while pirfenidone (*p* = 0.11) did not have an significant influence. The significant influence (*p* < 0.01) of the covariate of gender showed that men were significantly less likely to suffer from nausea than women.

As regards diarrhoea, nintedanib was associated with significantly increased (*p* < 0.01) diarrhoea complaints, while pirfenidone (*p* = 0.41) did not show any effect. Both nintedanib (*p* < 0.01) and pirfenidone (*p* < 0.01) users reported significantly reduced appetite, with women (*p* < 0.01) reporting significantly more appetite loss than men. Antacid use (*p* < 0.05) also increased appetite loss.

Weight loss appeared to be significantly higher among nintedanib users (*p* < 0.01) compared with non-drug users, while pirfenidone (*p* = 0.015) seemed to be associated with to have a less weight loss. A higher BMI was associated with a lower risk of weight loss (*p* < 0.05), whereas vitamin D use increased weight loss. Antacid use (*p* < 0.01) was also associated with significantly increased weight loss.

Finally, even though 31% of the respondents reported to suffer from a decrease in appetite or smell perception, Table 1 shows that this cannot be attributed to their drug use. Other covariates that tended to have an effect included gender (*p* = 0.01), as women were significantly more likely to suffer from loss of appetite or smell, and antacid use (*p* = 0.021), which increased the risk of loss of taste or smell perception.

## Discussion

Ideally, a progressive and almost invariably fatal disease like IPF should be treated, unless there is clear evidence of a lack of response. Benefits and burden of treatment should be discussed with every newly diagnosed IPF patient, taking his/her unique profile into account (Table 3). It is therefore important to gain more insights into the way the drugs work and their possible side effects. The current study found a difference in self-reported side effects between the two antifibrotic drugs nintedanib and pirfenidone among an IPF sample. In the majority of the cases, the use of the drug was continued despite the side effects. Among the 24 respondents who switched from pirfenidone to nintedanib, 21 mentioned side effects as an underlying reason. Among the five respondents switching from nintedanib to pirfenidone, two respondents mentioned side effects as an underlying reason.

**Table 3:** Patients’ comments in the survey: advice for prescribers

Prescribers should give patients guidance about taking medication to reduce side effects, such as what time of the day to take medication, or if it should be taken with food, and possible interactions. Hospital and/or community pharmacists could play a role, especially with regard to patients’ other possible drug use, e.g. statins
Prescribers should reassure patients about the variation in medication they are using, and inform them about possible interactions with other drugs, explaining why the combination is necessary
Prescribers should review any treatments prior to receiving the IPF diagnosis, such as long-term steroid use, and check for a risk of antibiotic resistance

Nintedanib users (48.3%) reported suffering from a significant increase in diarrhoea, weight loss, vomiting and loss of appetite, compared with non-drug users (11.4%). Pirfenidone users (40.3%) reported a significant increase in loss of appetite and weight loss. The degree of weight loss and loss of appetite did not differ significantly between the two groups of drug users. Other side effects reported by respondents in our study included dry mouth, dyspepsia, sun allergy and skin rash (data not shown), which was in line with a previous study by Bennet et al. [9].

Previous studies on the side effects of antifibrotic drugs have reported similar results. The nausea and vomiting associated with nintedanib (Fig. 1) was also found in previous research [10, 11]. By contrast, the association between pirfenidone and nausea or vomiting as found by previous studies was not confirmed by our results [12, 13]. A possible explanation could be that 68.1% of our respondents had used pirfenidone for more than 12 months, while most stomach complaints manifest within the first 3 months and decrease over time [14]. Furthermore, the problem of nausea can be reduced by taking pirfenidone immediately after food consumption [15].

The weight loss, loss of appetite and diarrhoea reported for nintedanib users in previous studies were in line with our current data, which also showed a significant prevalence [10, 11, 16, 17]. In order to counter these side effects, it could useful to look into dietary interventions. For example, the official nintedanib website recommends the Bananas Rice Applesauce Toast (B.R.A.T.) diet to counter diarrhoea [18].

Our pirfenidone users reported an increase in loss of appetite and weight loss, which is also in agreement with results from previous studies [13, 19, 20]. Finally, 31% of all respondents reported suffering from loss of taste or smell perception. Our data showed, however, that this was not caused by antifibrotic drug use. The loss of taste or smell perception could be influenced by covariates related to IPF. Women appeared more prone to loss of taste or smell perception than men, and antacid use also affected taste and smell perception. Another possible cause could be that the disease itself influences taste and smell perception. Reduced taste perception is also found in other lung diseases like COPD and lung cancer [21–24]. Lung function could play an important role in taste or smell perception. A possible underlying cause could be a relation between lung retention and retronasal smell perception, as suggested in a previous study [25]. Taste and smell perceptions are related and therefore retronasal smell perception could also influence taste. This process has also been suggested to play an important role in the flavour perception of vaping [25]. Another study found that lung retention, measured by the release of N-isopropyl-p[^123^I]-iodoamphetamine (^123^I-IMP) by the lung after ^123^I-IMP injection, was prolonged in lung fibrosis patients [26].

Besides knowing which side effects can occur due to the current treatment, it is also important to analyse whether this leads to drug discontinuation. In our study, drug discontinuation was rather rare. Two pirfenidone and one nintedanib user stopped their antifibrotic medication completely due to the side effects (data not shown).

In the ASCEND and CAPACITY studies, the reported pirfenidone side effects of skin rash, nausea and dyspepsia did not lead to drug discontinuation in the clinical trials [12, 13, 27]. Similarly, although more than 60% of patients receiving nintedanib experienced diarrhoea in the INPULSIS trials, this was often adequately controlled by dose reduction or anti-diarrhoeal medication, with < 5% of them having to discontinue the medication completely [28].

## Limitations

One of the limitations of this study is that information about disease severity was lacking, so the impact of disease severity on the side effects could not be established. Another limitation is that the symptoms were self-reported and not objectified by a health care professional.

## Recommendations

The data retrieved from our study show that both nintedanib and pirfenidone carry a high burden of gastrointestinal side effects. However, in line with real-life experiences which have clearly demonstrated that the gastrointestinal side effects rarely result in treatment discontinuation [29], only a few patients (n = 3) in the present study ultimately had to discontinue their medication. Therefore, it would be useful to look into possible dietary interventions to minimise this burden, as well as the use of other drugs to counter these side effects. It should be acknowledged that patients welcome supportive care throughout the trajectory of the disease, and patients should be supported at each step of the process. In this regard, accessible support from care providers, especially from ILD specialist nurses and nurse practitioners, plays a crucial role in shared decision making and handling gastrointestinal side effects of antifibrotics [30–32]. Strategies to manage gastrointestinal side effects caused by one of the antifibrotics start with the advice to take the tablets of both agents during a meal, not on an empty stomach, and to divide the dosage across the meal [33]. Thus, a reduction of the peak dose can be achieved by taking the medication with food [34]. In case of persistent diarrhoea, the next step may be rehydration and anti-diarrhoeal medication, for example, loperamide [35], and in case of persistent nausea, anti-emetics [36]. Furthermore, antacids are recommended in case of indigestion [33]. In addition to pirfenidone and nintedanib, there is also a conditional recommendation for proton-pump inhibitors in IPF treatment [37]. Because there is a risk of pharmacokinetic interaction between pirfenidone and omeprazole, this should be avoided when using pirfenidone, but may be given with nintedanib [30]. In contrast to what is known about pirfenidone and nintedanib, the data supporting the effect of antacid therapy in IPF are of poor quality (e.g. observational/retrospective studies and post hoc analysis of patients assigned to placebo arms in clinical trials of pharmaceutical interventions). The guidelines do acknowledge the need for further research on the efficacy and long-term safety of antacid therapy as well as interactions with other IPF medications.

**Fig. 2:**
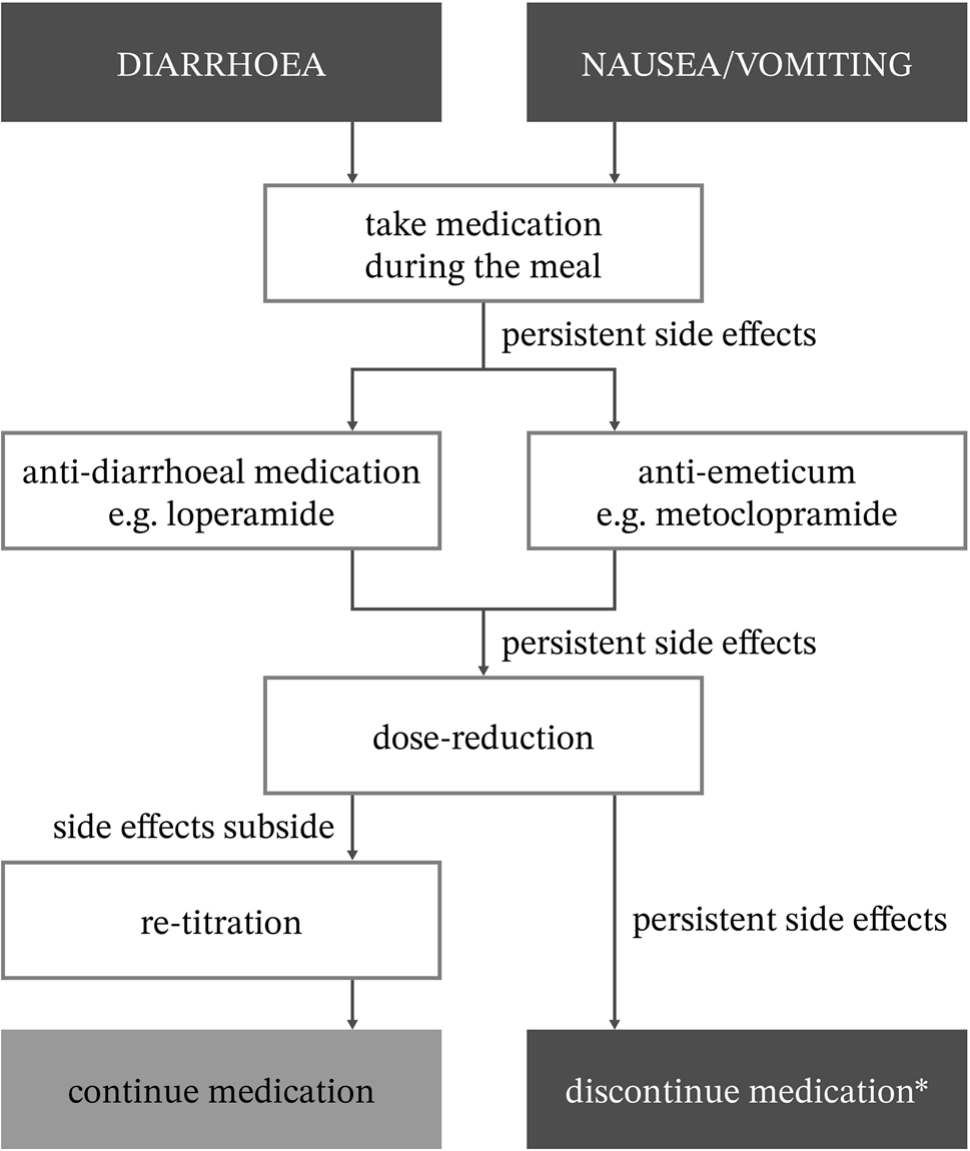
Algorithm for the management of gastrointestinal side effects of pirfenidone or nintedanib. *If a patient finds the side effects intolerable, and he/she really wants to stop. Optional: referral to a dietician and/or starting proton-pump inhibition

Although it is not ‘a one size fits all’ policy in case of persistent side effects, supervised dose reduction and re-titration may be required once the symptoms have subsided [30]. In case of weight loss, the decision depends on the patients’ own opinion. If the weight loss is more than 10%, patients should be referred to a dietician. An algorithm for the practical management of gastrointestinal side effects of pirfenidone or nintedanib, based on patients’ decisions and clinical practice, is presented in Fig. 2.

All authorised products of nintedanib and pirfenidone in the European Union are listed for close monitoring. These products are marked by regulatory authorities as requiring additional monitoring with regard to adverse drug reactions [38].

## Conclusion

Information about possible side effects is important if patients are to receive the best antifibrotic treatment available. The current study showed that the two antifibrotic drugs nintedanib and pirfenidone have different side effects.

Nintedanib users reported a significant increase in diarrhoea, vomiting, weight loss, and loss of appetite, while pirfenidone users suffered primarily from an increase in loss of appetite. In addition, nintedanib was associated with nausea and pirfenidone with weight loss.

Our data showed that 24 respondents had switched from pirfenidone to nintedanib in the past, while five had switched from nintedanib to pirfenidone, suggesting that although nintedanib gave rise to more gastrointestinal side effects, the general burden of side effects of nintedanib is probably lower.

Both pirfenidone and nintedanib carry a rather high burden of gastrointestinal side effects, so it could be useful to look into dietary interventions to minimise this burden.

## Data Availability

The datasets used and/or analysed during the current study are available from the corresponding author on reasonable request.

## Appendix

See Table 4.

**Table 4:** Correlation matrix used to define covariates

	Nausea	Vomiting	Diarrhoea	Weight loss	Appetite loss	Loss taste/smell
Age	− 0.04	− 0.03	− 0.14	0.02	0.04	0.01
Gender	− 0.33**	− 0.26**	0.12	− 0.03	− 0.22**	− 0.25**
BMI	0.00	− 0.05	0.01	− 0.20**	− 0.11	− 0.07
Time since diagnosis	− 0.06	− 0.01	0.07	0.06	0.05	0.19*
Smoking	0.02	0.20**	0.09	0.09	0.06	− 0.11
Vit.D	0.13	0.09	− 0.14	0.19*	0.11	0.07
Vit.K	0.07	0.06	− 0.12	0.08	0.08	0.06
Antacid	0.10	0.01	− 0.14	0.27**	0.18*	0.19*
Multivit	0.02	− 0.06	− 0.11	− 0.11	− 0.06	− 0.01
Oxygen use	0.04	0.08	0.08	− 0.07	− 0.07	− 0.03

If a *p* value was lower than 0.05, the covariate was included

^*^*p* < 0.05; ***p* < 0.01
